# Effect of SNS addiction on prosocial behavior: mediation effect of fear of missing out

**DOI:** 10.3389/fpsyg.2024.1490188

**Published:** 2024-12-23

**Authors:** Manjing Xu, Donglin Liu, Jinzhe Yan

**Affiliations:** ^1^Business School, Zhengzhou University of Economics and Business, Zhengzhou, China; ^2^Department of Business Administration, Gachon University, Seongnam, Republic of Korea; ^3^College of Business, Gachon University, Seongnam, Republic of Korea

**Keywords:** networking sites addiction, fear of missing out, basic psychological need satisfaction, prosocial behavior, SNS addiction

## Abstract

**Purpose:**

This study examines the relationship between social networking sites addiction and pro-social behavior, considering the increasing importance of social networking sites in daily life. It explores the mediating role of Fear of Missing Out in this relationship and investigates the moderating role of basic psychological need satisfaction.

**Research design, data, and methodology:**

This study employed a snowball sampling method to conduct an online survey among social network users in China. The proposed model was tested using regression analysis to interpret the results.

**Results:**

Findings indicate a negative predictive effect of social networking sites addiction on prosocial behavior. Misplaced fear partially mediates this relationship. Basic psychological need satisfaction significantly moderates the mediating effect of Fear of Missing Out on the relationship between SNS addiction and prosocial behavior.

**Practical implications:**

This study provides strategies for effectively preventing social networking sites addiction in real-world settings and mitigating its negative impact on individuals’ physical and mental health. It suggests interventions at four levels—individual, school, society, and government—to enhance basic psychological need satisfaction, thereby improving prosocial behavior and facilitating the promotion of interpersonal interactions and the equitable, harmonious development of society.

## 1 Introduction

The China Internet Network Information Center (CNNIC) released the 53nd Statistical Report on Internet Development in China (China Internet Network Information Center, 2023). The Report indicates that as of December 2023, China’s Internet users numbered 1.092 billion, with 24.8 million new netizens compared to December 2022, and the Internet penetration rate reached 77.5%. The Internet has become vital for entertainment, education, communication, and information sharing. With advancements in Internet technology, social networking sites (SNS) have become integral to our daily lives (Yin et al., 2021). Social network users exchange content such as videos and pictures, engage in chats, and expand their communication with a broader audience through posts, texts, and comments via networks like Facebook, Twitter, and Instagram (Huda, 2022).

While SNS offer numerous benefits, such as improved communication, social connections, enjoyment, and interaction (Chatterjee et al., 2022), and help students in their educational pursuits (Anshari et al., 2017), the increased use of these technologies has led to several adverse behaviors and outcomes like reduced productivity and deteriorated physical and mental health (Yang et al., 2016). Unrestricted interaction on social networks can raise ethical concerns leading to issues like privacy breaches from exposure of personal data, security risks from public systems, defamation, and the spread of harmful content (Li, 2015). The rising prevalence of cyberbullying among social network users serves as an alarm (Moore and Craciun, 2021). Moreover, research indicates that SNS-addicted users are more likely to show clinical signs of psychiatric disorders such as depression and schizophrenia compared to non-addicts (Wang et al., 2023). Negative emotions can also coincide with dysfunctional social interactions, resulting in psychological and physiological responses that may trigger harmful behaviors (Garland et al., 2010). Turkle (2016) proposes that excessive reliance on technology impairs social skills, hindering meaningful interactions and leading to diminished attention spans and retention worsened by constant connectivity. Oh (2012) discovered that increased addiction to social networks often correlates with higher levels of loneliness and depression and deteriorates satisfaction with relationships. Problematic social network use (PSNU) is marked by symptoms like loss of control, obsession, withdrawal, and emotional dysregulation, negatively impacting social activities, interpersonal relationships, and well-being (Bányai et al., 2017). Personal distress tends to foster egocentric behavior and moral disengagement rather than encouraging prosocial behavior (Paciello et al., 2013). The effects of SNS addiction on prosocial behavior, and the underlying conditions and mechanisms remain underexplored.

The intensity of social media use is significantly and positively correlated with Fear of Missing Out (Buglass et al., 2017). Both the use of multiple social media platforms and problematic social media use significantly impact Fear of Missing Out as well (Primack et al., 2017). Fear of Missing Out has been recognized as an important psychological construct associated with increased use of SNS and technology (Elhai et al., 2020). Anxiety is a critical component of Fear of Missing Out (FoMO), and anxiety-induced negative emotions can adversely affect an individual’s social status or factors related to social status (Beyens et al., 2016). Research indicates that anxiety and prosocial behavior are negatively correlated (Duchesne et al., 2010). According to cognitive resource theory, anxiety is a complex emotional response that consumes substantial attentional resources. Consequently, when an individual’s attention is focused on their own anxiety, they invest fewer external attentional resources, and their willingness to help others decreases. The relationship between FoMO, SNS addiction, and prosocial behavior requires further exploration.

A high level of basic psychological need satisfaction positively influences an individual’s healthy physical and mental development and behavioral performance (Burgueño et al., 2023; Adam et al., 2023; Rowicka and Postek, 2023). Conversely, dissatisfaction or low satisfaction of basic psychological needs, also known as basic psychological satisfaction frustration, leads to negative and unhealthy emotional and behavioral manifestations (Liu et al., 2017; Gu et al., 2023). The regulatory role of basic psychological need satisfaction on the effects of FoMO on prosocial behavior in the context of SNS addiction remains underexplored.

Previous studies in the literature have primarily explored the causes, influencing factors, and negative consequences of SNS addiction (Xu et al., 2022; Miranda et al., 2023; Kwak and Kim, 2023; Lee et al., 2023), yet there are some limitations. Firstly, the impact of SNS addiction on prosocial behavior has not been adequately addressed. Secondly, the mechanisms and conditions under which SNS addiction influences prosocial behavior require further investigation. To address these research gaps, the main objectives of this study are: first, to analyze the effects of SNS addiction on prosocial behavior; second, to explore how FoMO mediates the effects of SNS addiction on prosocial behavior; and third, to examine how basic psychological need satisfaction mediates the effects of FoMO on prosocial behavior in situations of SNS addiction.

The structure of the paper is summarized as follows: after the initial introduction, the literature review and relevant hypotheses are presented. This is followed by the research methodology. Subsequently, the findings, discussion, and implications of the study are detailed. Lastly, the study’s limitations and future perspectives are outlined.

## 2 Literature review and hypothesis

### 2.1 SNS addiction and prosocial behavior

Social network site (SNS) addiction is described as excessive focus on these platforms, propelled by an uncontrollable urge to log on or use these sites, consuming substantial time and energy, and leading to uncontrollable problematic behaviors. These behaviors produce physical and psychological problems and impair other important life areas (Błachnio et al., 2016).

SNS addiction represents a maladaptive psychological reliance on social networks, significantly negatively impacting important user life areas (Qahri-Saremi et al., 2021). Research indicates that SNS addiction may provoke anxiety, depressive symptoms, mental fatigue, physical illness, guilt, cyber-violence, decreased sleep quality, and even suicidal intent, severely threatening physical and mental health (Jiang et al., 2016; Turel, 2016; Sampasa-Kanyinga and Hamilton, 2015). Children facing social family adversity, inattention, and low classroom pro-sociality are at a heightened risk for severe anxiety symptoms (Duchesne et al., 2010). Negative emotions such as depression and anger correlate with prosocial behavior. An experimental study demonstrated that expressions of disappointment enhance adherence to help-seeking, while expressions of anger reduce it (Van Doorn et al., 2015). According to Cognitive Resource Theory and Attentional Resource Theory, emotions like sadness, anxiety, or depression impact behaviors through individual cognition and limit prosocial behavior by consuming the individual’s finite attentional resources. This distraction makes the individual less aware of external surroundings. If these emotions are unregulated, sadness and anxiety can decrease prosocial behavior (Eisenberg, 2000). The social displacement hypothesis (Kraut et al., 1998) posits that increased online communication leads to decreased offline communication, promoting social withdrawal and diminished problem-solving skills.

Prosocial behavior involves the voluntary, cooperative effort of helping others, often at personal expense, for the benefit of others. This includes actions such as helping, sharing, donating, comforting, cooperating, and volunteering (Snippe et al., 2018). Prosocial behavior comprises both helping behavior, which involves providing aid to others, and altruistic behavior, which entails helping others without expecting any return. Prosocial behavior not only benefits the recipient but also enhances the helper’s well-being and life satisfaction (Yang et al., 2016). It also fosters better interpersonal interactions and promotes the harmonious development of social equity (Penner et al., 2005). Consequently, this study proposes the following hypothesis:

*H1*: SNS addiction negatively affects prosocial behavior.

### 2.2 The mediating role of fear of missing out

Individuals using Facebook for extended periods may perceive life as unfair and believe that others lead happier, more fulfilling lives (Chou and Edge, 2012). This perception contributes to a generalized anxiety that others may be experiencing positive events from which one is excluded. FoMO, or Fear of Missing Out, is an anxiety stemming from the fear that one is missing out on the positive experiences of others; it is defined as a stable personality trait where individuals have a strong expectation to consistently monitor others’ activities (Przybylski et al., 2013). Those experiencing FoMO manifest diffuse anxiety emotionally, have a strong cognitive expectation to keep up with others, and often engage in maladaptive social media behaviors, failing to meet their emotional, cognitive, and behavioral needs. According to self-regulation theory, impaired self-regulation in individuals leads to FoMO (Chai et al., 2018).

The study revealed that a positive relationship exists between SNS addiction and adolescent FoMO, with jealousy mediating this relationship (Yin et al., 2021). Problematic smartphone use was highly linked with anxiety, need for touch, and FoMO, with frequency of use inversely related to depression (Elhai et al., 2016). There was a significant correlation between SNS use and FoMO, with increased Facebook use indicating higher FoMO levels (Buglass et al., 2017). Moreover, frequent use of internet communication applications might trigger or augment an aspect of FoMO specifically related to the online activities of other users, which arises in the more volatile context of internet communication (Wegmann et al., 2017). Additionally, excessive SNS usage has been shown to result in high levels of FoMO among users (Przybylski et al., 2013). Therefore, this study proposes the following hypothesis:

*H2*: SNS addiction positively affects FoMO.

According to the Image Management Theory (Siibak, 2009), SNS provide a platform for individuals to reconstruct their personal image based on desirable self-identification values, which leads to the prevalence of people uploading information that showcases their lavish and interesting activities, and viewing large amounts of such information can easily lead to social comparisons. According to Social Comparison Theory, in upward social comparisons, people tend to evaluate and compare themselves to those who are better than them in some relevant areas (Appel et al., 2016), and the results of such comparisons can lead people to believe that other people are better off than they are, which can lead to a “falling behind” feeling and threatens their self-concept, meaning that people with more problematic SNS use are more likely to experience FoMO (Yin et al., 2023). According to feelings-as-information theory, individuals tend to use their feelings as sources of information. Sadness, which conveys a ‘problematic’ situation, motivates individuals to concentrate on details and is accompanied by decreased amusement (Schwarz, 2002). Both FoMO and problematic internet use can negatively affect subjective well-being beyond personality (Stead and Bibby, 2017), resulting in reduced physical satisfaction, psychological well-being, and helping behaviors (Buglass et al., 2017). Studies have demonstrated that anxiety behavior does not significantly correlate with kindergarten achievement, whereas prosocial behavior does (Collie et al., 2018). Cognitive Resource Theory and Attentional Resource Theory suggest that due to the limited and selective nature of attentional resources, individuals with FoMO are likely to focus more on personal-relevant matters, allocate fewer attentional resources to unrelated matters, easily overlook others’ situations, and exhibit reduced prosocial behavior (Xia et al., 2021). Therefore, this study proposes the following hypothesis:

*H3*: FoMO negatively affects prosocial behavior.

*H4*: FoMO mediates the relationship between SNS addiction and prosocial behavior.

### 2.3 The moderating role of basic psychological need satisfaction

Drawing upon Self-Determination Theory (SDT), basic psychological needs of human beings encompass autonomy, competence, and relatedness (Deci and Ryan, 2008). Basic psychological need satisfaction serves as a psychological variable that significantly elucidates the causal mechanisms driving the impact of environmental factors on individuals’ psychological and behavioral inclinations. Human individuals are born with a drive for growth and development; thus, they strive to harmonize external environmental factors with their self-concept. This innate drive is contingent on the fulfillment of the three fundamental psychological needs (Deci and Ryan, 2008).

Drawing on Self-Determination Theory (SDT) and the dichotomous model of passion, high levels of need satisfaction do not predict playtime, yet they correlate with increased harmonious passion, playfulness, and post-play energy (Przybylski et al., 2009). Individuals engaging in prosocial behavior for pleasure report higher life satisfaction, more positive emotions, and fewer negative emotions compared to those driven by pressures of responsibilities (Gebauer et al., 2008). An essential source of individual well-being is the satisfaction of basic psychological needs (Ryan et al., 2008), which consistently predicts well-being. Individuals focused on meaningful happiness prioritize personal growth, which enhances their capacity for autonomous aid and connectivity with others (Ryan et al., 2008). Satisfying basic psychological needs significantly mitigates the impact of SNS addiction on prosocial behavior.

When basic psychological needs remain unmet, individuals seek psychological satisfaction through compensatory behaviors and substitutes (Deci and Ryan, 2000). Empirical research indicates that individuals failing to satisfy basic psychological needs in real-life settings are prone to psychological distress, which heightens the risk of Internet addiction (Wong et al., 2015) and can lead to increased aggression as another substitute for unmet needs (Kuzucu and Şimşek, 2013). Lower satisfaction of basic psychological needs amplifies the negative effects of SNS addiction on prosocial behavior. Accordingly, this study posits the following hypothesis:

*H5-1*: Autonomy moderates between the effects of SNS addiction on prosocial behavior.

*H5-2*: Competence moderates between the effects of SNS addiction on prosocial behavior.

*H5-3*: Relatedness moderates between the effects of SNS addiction on prosocial behavior.

When the external environment fulfills an individual’s basic psychological needs, it fosters their natural inclinations toward self-integration, establishes positive and harmonious emotional relationships, enhances well-being, and facilitates better adaptation to social changes (Przybylski et al., 2009). It also alleviates anxiety and depression, playing a crucial role in healthy psychological development and well-being (Deci and Ryan, 2000). Individuals who report high levels of satisfaction with autonomy needs, competence needs, or relationship needs are better at regulating their emotions and report fewer mental health problems (Moè and Katz, 2021). Basic psychological needs satisfaction can enhance an individual’s positive emotions and inhibit their negative emotions (Deci and Ryan, 2004; Ryan and Deci, 2008). Research has confirmed a significant negative correlation between basic psychological needs (autonomy, competence, and relatedness needs) satisfaction and levels of FoMO (Przybylski et al., 2013), with adolescents with high levels of basic psychological needs satisfaction perceiving lower levels of FoMO, whereas adolescents with deficits in basic psychological needs satisfaction perceive higher levels of FoMO (Oberst et al., 2017; Alt, 2015; Beyens et al., 2016). Various forms of interpersonal insecurity can inhibit or interfere with prosocial behavior when prone to self-doubt, anxiety, social fears, and other psychological problems (Mikulincer and Shaver, 2005; Twenge et al., 2007). High basic psychological needs satisfaction attenuates the mediating role of FoMO between SNS addiction and prosocial behavior.

According to Self-Determination Theory, autonomy, competence, and relatedness belong to basic psychological needs, and subordination, socialization, belonging, hedonic, material, and information belong to other needs, and the degree of psychological needs satisfaction affects the degree of FoMO, and those with low psychological needs satisfaction have a higher degree of FoMO (Swar and Hameed, 2017). Przybylski et al. (2013) found that individuals with lower levels of satisfaction of basic psychological needs such as competence (efficacy), autonomy (meaningful choices), and relatedness (connection with others) reported higher levels of FoMO, and that deficits in basic psychological needs may increase sensitivity to the fear of missing out on things, which in turn may drive people to use social media, as social media can provide individuals with effective self-regulatory tools to fulfill their psychological needs. Individuals who do not perceive basic psychological need satisfaction in their environment develop negative emotional and behavioral tendencies (Murphy et al., 2023). When an individual’s basic psychological needs are thwarted, depressive tendencies tend to emerge. Compared to non-depressed adults, individuals with depression exhibit higher levels of frustration and lower satisfaction in meeting the three basic psychological needs (Pietrek et al., 2022). When an individual’s basic psychological needs satisfaction is not met, he or she will focus on his or her own needs and will not pay attention to the surrounding environment, which may lead the individual to follow external rules, which may make the individual’s behavior unmotivated or even engage in antisocial behaviors (Deci and Ryan, 2000). Basic psychological need satisfaction is the internal motivation that drives individuals to engage in compensatory behaviors when their needs are unmet (Sheldon et al., 2011). Consequently, FoMO individuals deplete attentional resources, are more likely to disregard reality, thus increasing perceived interpersonal distance and diminishing psychological well-being (Diener, 1984), and reduce prosocial behavior. Low basic psychological needs satisfaction enhances the mediating role of FoMO between SNS addiction and prosocial behavior. Therefore, this study proposes the following hypothesis:

*H6-1*: Autonomy moderates the mediating effect of FoMO.

*H6-2*: Competence moderates the mediating effect of FoMO.

*H6-3*: Relatedness moderates the mediating effect of FoMO.

**Figure 1:**
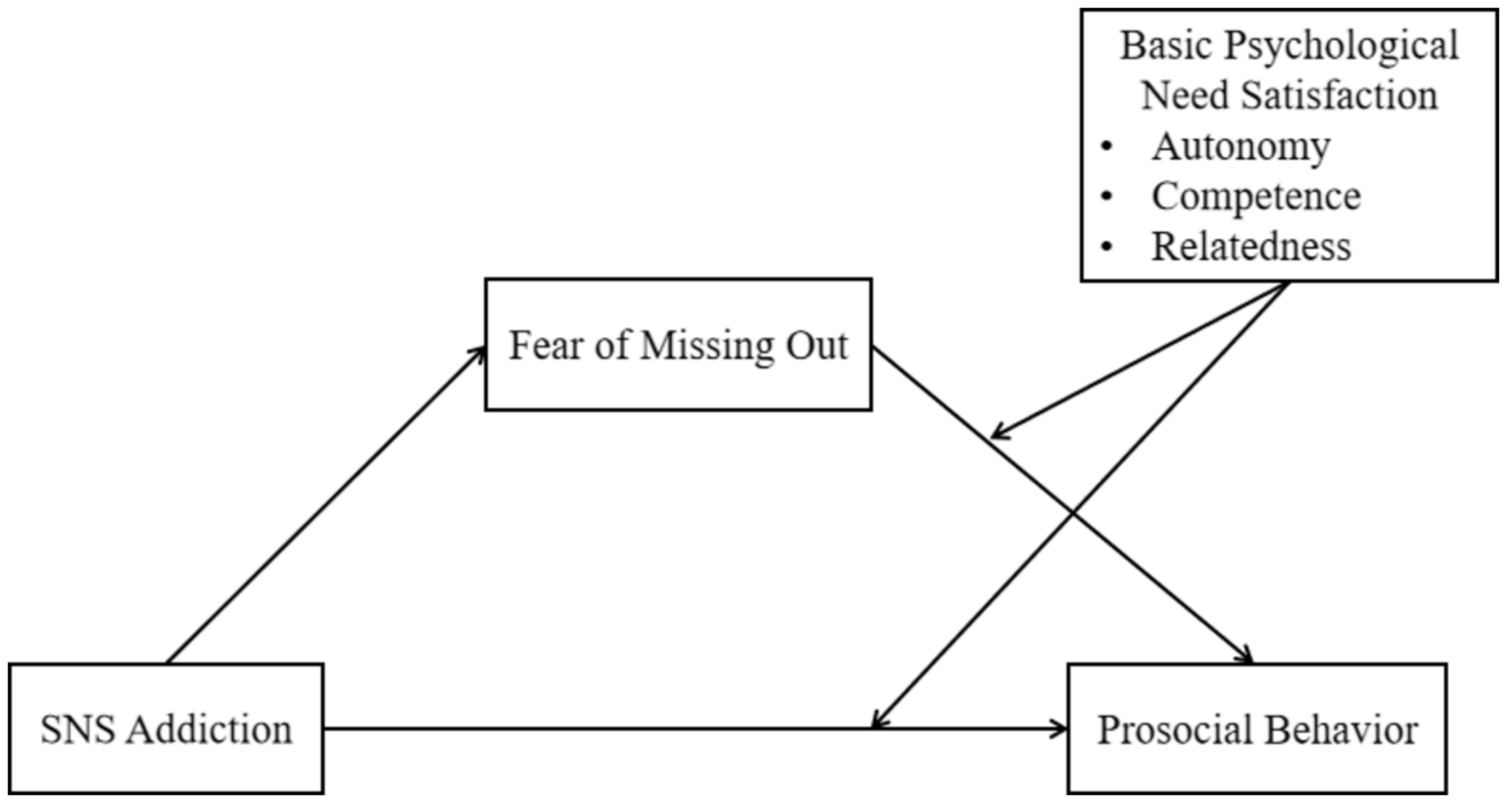
Research model.

In summary, this study aims to explore the relationship between SNS addiction, FoMO, basic psychological need satisfaction, and prosocial behavior, along with their mechanisms of action, and to propose a research model (see Figure 1).

## 3 Research methodology

### 3.1 Data collection and sample

We conducted an online survey among social network users in China. Before the formal survey, a pilot test was conducted with 40 SNS users. Measurement items and questionnaires were refined based on participants’ feedback. After launching the formal survey, participants were recruited through Snowball Sampling (Przepiorka and Blachnio, 2016). Online survey participants were informed that the study was related to SNS activities and were asked to distribute the questionnaire link to their social network friends, inviting them to participate. Participants were explicitly assured in the questionnaire that their responses would be treated confidentially and anonymously. Additionally, participants were advised of their freedom to participate or withdraw from the study at any time.

Finally, 670 questionnaires were returned; after removing invalid ones, 624 valid questionnaires were collected, reflecting a recovery rate of 93.13%. The age range of the sample was 14–60 years old, with 52.6% females, M_age_ = 28.44 years, SD = 9.35. Detailed demographic information about our sample is presented in Table 1.

**Table 1:** Demographic information of respondents (*N* = 624).

Demographics	Classification	Frequency	Percentage
Gender	Male	296	47.4
	Female	328	52.6
Age	Under 19	135	21.6
	20–29	233	37.3
	30–39	157	25.2
	40–49	75	12.0
	Above 50	24	3.9
Education	High school or below	92	14.7
	Three-year college	224	35.9
	Four-year college	263	42.1
	Graduate school or above	45	7.2
Career	Students	192	30.8
	Foreign enterprise	38	6.1
	Private enterprise	187	30.0
	State-owned enterprise or institution	34	5.4
	Civil servant	28	4.5
	Self-employed	72	11.5
	Other	73	11.7
Income	¥2,000 and below	173	27.7
	¥2001 – ¥4,000	89	14.3
	¥4,001 – ¥6,000	149	23.9
	¥6,001 – ¥8,000	90	14.4
	¥8,001 – ¥10,000	65	10.4
	¥10,001 and above	58	9.3

### 3.2 Measures

#### 3.2.1 SNS addiction

Andreassen (2015) defines SNS addiction as “excessive attention to SNS, being driven by a strong motivation to log on to or use SNS, and investing excessive time and energy in SNS to the detriment of other social activities, school/work, relationships, mental health and well-being.” The SNS Addiction Scale, adapted by Gao et al. (2017) and revised by marketing and English language professionals through back-translation, includes six items (e.g., “It is difficult for me to concentrate on my studies or work due to the use of social networks”) to assess SNS addiction. Each item is rated on a 5-point scale (1 = “very rarely,” 5 = “frequently”), suggesting higher scores indicate more severe SNS addiction. The Cronbach’s *α* for this scale was 0.886, affirming its reliability and appropriateness for Chinese samples.

#### 3.2.2 Fear of missing out

FoMO is a personality construct and process defined by an unwillingness to miss out on important experiences and a desire to stay connected to one’s social network (Przybylski et al., 2013). FoMO was assessed using the Fear of Missing Out Scale by Przybylski et al. (2013), which comprises 10 items (e.g., “I am afraid that others are more productive than I am”). Each item is rated on a 5-point scale (1 = “not at all true for me,” 5 = “Extremely true of me”), structured around a single factor, where a higher score denotes more severe FoMO. The scale was adapted for Chinese cultural context and language preferences, achieving a Cronbach’s *α* of 0.936, which confirms the scale’s reliability in this version.

#### 3.2.3 Basic psychological need satisfaction

Self-Determination Theory suggests that humans are born with three basic psychological needs – autonomy, competence, and relatedness (Deci and Ryan, 2000). Autonomy is the basic psychological needs satisfaction of having choice, a sense of will, and approval over one’s behavior (Baard et al., 2004), and when individuals have a sense of will and control over their lives (Ryan and Deci, 2017). Competence is related to a person’s feeling of success in completing tasks that are optimally challenging and personally meaningful, as well as a sense of validity, and an individual’s need for competence is met when they experience a sense of being able to bring about the desired change in their environment (Ryan and Deci, 2000a; Ryan and Deci, 2000b). Relatedness is related to one’s connection to others in the group, and this need is met when people feel respected and valued by others (Ryan and Deci, 2000a; Ryan and Deci, 2000b). The Basic Psychological Need Satisfaction Scale by Johnston and Finney (2010) assesses three core needs: autonomy, competence, and relatedness, through a 21-item scale (e.g., “I feel that I have the freedom to make decisions about how to live my life”). Each item uses a 5-point scale (1 = “not at all true for me,” 5 = “very true for me”). The Cronbach’s α coefficients stood at 0.896, 0.874, and 0.916 for autonomy, competence, and relatedness respectively, indicating the scale’s high reliability for Chinese audiences.

#### 3.2.4 Prosocial behavior

Altruistic behavior, as a specific form of prosocial behavior, refers to behavior that reduces one’s own survival fitness in order to help other individuals with the primary goal of helping others (Schwartz et al., 2012). Prosocial behavior was evaluated using the Altruistic Prosocial Behavior Scale developed by Carlo and Randall (2002), consisting of 5 items (e.g., “I think one of the best things about helping others is that it makes me look good”). Each item uses a 5-point scale (1 = “not at all,” 5 = “fully”), with a Cronbach’s α of 0.902, indicating good reliability and suitability for use with Chinese samples. All the measurement items refer to Supplementary files.

### 3.3 Statistical analysis

First, descriptive statistics and Pearson correlations among the study variables were calculated. Second, the PROCESS macro of SPSS (Model 4) was utilized to assess the mediating effect of FoMO (Hayes, 2012). Finally, the PROCESS macro (Model 15) was used to examine the moderating role of basic psychological need satisfaction in the direct and indirect relationships between SNS addiction and prosocial behavior. We assessed the significance of the effects using bootstrap methods to achieve robust standard errors for the parameter estimates (Hayes, 2017). The bootstrap methods yielded 95% bias-corrected confidence intervals for these effects from 5,000 data resamples. Confidence intervals that exclude zero indicate statistically significant effects.

### 3.4 Common method bias test

A common method variance test was conducted using the Harman one-way test, along with an exploratory factor analysis for all quiz questions in the study. It was determined that the cumulative explained variance was 64.419%, and the largest common factor accounted for only 32.032% of the total variance, remaining under the critical threshold of 40% (Podsakoff et al., 2003), suggesting that the study was not severely affected by common method bias.

### 3.5 Reliability and validity tests

The present study followed the guidelines of Fornell and Larcker (1981) to assess the reliability and validity of the scale. We evaluated the factor loadings (LOF), reliability (CR), average variance extracted (AVE), and Cronbach’s alpha (CA). The analysis confirmed that all variables achieved the required thresholds for reliability and validity, as presented in Table 2, CR_LL_ > 0.8 (0.876_LL_-0.936_UL_), AVE_LL_ > 0.5 (0.544_LL_-0.653_UL_), CA_LL_ > 0.8 (0.874_LL_-0.936_UL_), indicating that all measurements are valid. This study performed a confirmatory factor analysis (CFA) in AMOS 24.0 to validate the applicability of the proposed measurement model. For this purpose, six factors (SNS addiction, FoMO, autonomy, competence, relatedness, and prosocial behavior) were interrelated and evaluated through CFA. The CFA outcomes indicated that the proposed measurement model fits acceptably. The model fit threshold metrics recommended by Kline (2023) were met and the structural model metrics fit the data well (Table 2).

**Table 2:** Confirmatory factor analysis.

Variables	Term	Loading	CR	AVE	α
SNS addiction	SNSA1	0.719	0.887	0.566	0.886
	SNSA2	0.741			
	SNSA3	0.752			
	SNSA4	0.738			
	SNSA5	0.756			
	SNSA6	0.806			
FoMO	FoMO1	0.787	0.936	0.597	0.936
	FoMO2	0.763			
	FoMO3	0.736			
	FoMO4	0.714			
	FoMO5	0.737			
	FoMO6	0.719			
	FoMO7	0.759			
	FoMO8	0.751			
	FoMO9	0.826			
	FoMO10	0.914			
Autonomy	AUT1	0.778	0.897	0.554	0.896
	AUT2	0.735			
	AUT3	0.799			
	AUT4	0.74			
	AUT5	0.697			
	AUT6	0.714			
	AUT7	0.74			
Competence	COM1	0.855	0.876	0.544	0.874
	COM2	0.774			
	COM3	0.675			
	COM4	0.64			
	COM5	0.721			
	COM6	0.741			
Relatedness	REL1	0.807	0.917	0.580	0.916
	REL2	0.78			
	REL3	0.737			
	REL4	0.767			
	REL5	0.686			
	REL6	0.767			
	REL7	0.776			
	REL8	0.766			
Prosocial behavior	PB1	0.803	0.904	0.653	0.902
	PB2	0.79			
	PB3	0.777			
	PB4	0.813			
	PB5	0.856			
Model Fit Index	χ^2^/df = 1.349, RMSEA = 0.024, NFI = 0.934, GFI = 0.926, CFI = 0.982, IFI = 0.982, TLI = 0.981, RMR = 0.040, SRMR = 0.03, AGFI = 0.917				

α = Cronbach’s alpha; AVE, Average Variance Extracted; CR, Construct Reliability. All loadings and estimates are significant at *p* < 0.001.

The discriminant validity of the scale was assessed and descriptive statistics were obtained using the method of Fornell and Larcker (1981). The results demonstrated that the square root of the AVE for each variable was greater than the interrelationships among the study variables, confirming good discriminant validity of the scale (Table 3).

**Table 3:** Discriminant validity and descriptive statistics.

	M (SD)	1	2	3	4	5	6
1. SNS Addiction	3.750 (0.956)	**0.752**					
2. FoMO	3.489 (0.969)	0.586**	**0.773**				
3. Autonomy Need	3.514 (0.935)	−0.179**	−0.238**	**0.744**			
4. Competence Need	3.714 (0.833)	−0.190**	−0.309**	0.417**	**0.738**		
5. Relatedness Need	3.578 (0.898)	−0.300**	−0.336**	0.390**	0.337**	**0.762**	
6. Prosocial Behavior	3.603 (1.032)	−0.567**	−0.599**	0.447**	0.425**	0.483**	**0.808**

*p < 0.05, **p < 0.01, ***p < 0.001. M: Mean; SD: Standard deviation.

## 4 Empirical results

### 4.1 Hypothesis testing

Regression analysis was conducted using SPSS to examine the relationship between variables in this study. The results indicated (Table 4) that SNS addiction significantly and negatively impacts prosocial behavior (*β* = −0.567, Boot = [−0.681, −0.541]). The validity of Hypothesis 1 is confirmed as the confidence interval for the test results does not include 0, suggesting that SNS addiction undermines prosocial behavior. SNS addiction also significantly enhances FoMO (β = 0.586, Boot = [0.529,0.658]). Since the confidence interval does not embrace 0, Hypothesis 2 is upheld, indicating that SNS addiction contributes positively to FoMO. Furthermore, FoMO significantly detracts from prosocial behavior (β = −0.599, Boot = [−0.705, −0.571]), supporting Hypothesis 3, as the confidence interval excludes zero, pointing out FoMO’s adverse effect on prosocial behavior.

**Table 4:** Path test results.

Relationships	S.E	β	*t*	BootULCI	BootLLCI	Results
H1:SNS Addiction→Prosocial Behavior	0.036	−0.567	−17.158	−0.681	−0.541	Supported
H2:SNS Addiction→FoMO	0.033	0.586	18.037	0.529	0.658	Supported
H3:FoMO→Prosocial Behavior	0.034	−0.599	−18.663	−0.705	−0.571	Supported

FoMO, Fear of Missing Out; S.E, Standard Error; β, Standardized Coefficient; *t* = tvalue. All relationships are significant *p* < 0.001.

### 4.2 Mediation analysis

The PROCESS macro of SPSS (Model 4) was utilized to assess the mediating effect of FoMO (Hayes, 2012). Regression analyses demonstrated that initially, SNS addiction negatively predicts prosocial behavior, β = −0.557, *p* < 0.001 (refer to Model 1 in Table 5). Subsequently, SNS addiction positively predicts FoMO, β = 0.585, *p* < 0.001 (refer to Model 2 in Table 5). In the final step, when controlling for SNS addiction, FoMO significantly negatively influences prosocial behavior, *β* = −0.405, *p* < 0.001 (refer to Model 3 in Table 5). The bias-corrected percentile bootstrap method reveals a significant indirect effect of SNS addiction on prosocial behavior via FoMO, ab = −0.256, SE = 0.022, 95% CI = [−0.285, −0.196], accounting for 42.537% of the total effect. This suggests that FoMO partially mediates the relationship between SNS addiction and prosocial behavior, confirming the validity of Hypothesis H4.

**Table 5:** Mediation model tests for fear of missing out.

Predictors	Model1 (prosocial behavior)	Model 2 (FoMO)	Model3 (prosocial behavior)
SNS Addiction	−0.557*** −16.714	0.585*** 17.758	−0.320*** −8.519
FoMO			−0.405*** −10.843
R^2^	0.330	0.345	0.438
AdjustedR^2^	0.324	0.338	0.431
F	50.716***	54.120***	68.480***

*p < 0.05, **p < 0.01, ***p < 0.001.

### 4.3 Moderating effect

The moderating role of the need for autonomy on the mediating effect of FoMO was analyzed in SPSS using the PROCESS macro (Model 15) (Hayes, 2012). The study revealed that the need for autonomy significantly moderated the relationship between SNS addiction and prosocial behavior. Specifically, a notable simple effect was observed when the need for autonomy was low (simple effect = −0.354, SE = 0.048, 95% CI [−0.448, −0.259], interval not including 0). Conversely, when the need for autonomy was high, the simple effect remained significant (simple effect = −0.218, SE = 0.051, 95% CI [−0.317, −0.119], interval does not contain 0), confirming hypothesis H5-1. Additionally, the need for autonomy influenced the FoMO-prosocial behavior relationship by moderating SNS addiction, with significant simple effects noted at both low (simple effect = −0.262, SE = 0.041, 95% CI [−0.338, −0.177], interval not containing 0) and high levels of autonomy (simple effect = −0.154, SE = 0.025, 95% CI [−0.202, −0.106], interval does not contain 0), thereby establishing hypothesis H6-1 (Table 6).

**Table 6:** The moderating effect of autonomy.

Relationships	AUT	Effect	SE	LLCI	ULCI
SA → PB	Low (2.579)	−0.354	0.048	−0.448	−0.259
	Middle (3.514)	−0.286	0.037	−0.359	−0.213
	High (4.449)	−0.218	0.051	−0.317	−0.119
SA → FoMO →PB	Low (2.579)	−0.262	0.041	−0.338	−0.177
	Middle (3.514)	−0.208	0.025	−0.256	−0.158
	High (4.449)	−0.154	0.025	−0.202	−0.106

SE, Standard Error; SA, SNS Addiction; FoMO, Fear of Missing Out; AUT, Autonomy; PB, Prosocial Behavior.

The moderating role of the need for competence on the mediating effect of FoMO was assessed in SPSS using the PROCESS macro (Model 15) (Hayes, 2012). It was found that the need for competence moderates the relationship between SNS addiction and prosocial behavior with a significant simple effect when the need for competence was low (simple effect = −0.390, SE = 0.044, 95% CI [−0.475, −0.305], interval not including 0), confirming hypothesis H5-2. Furthermore, the need for competence also influenced the FoMO-prosocial behavior relationship through its moderating effects on SNS addiction, with marked simple effects at both low (simple effect = −0.233, SE = 0.039, 95% CI [−0.313, −0.160], interval not containing 0) and high levels of competence (simple effect = −0.131, SE = 0.028, 95% CI [−0.183, −0.074], interval does not contain 0), thereby establishing hypothesis H6-2 (Table 7).

**Table 7:** The moderating effect of competence.

Relationships	COM	Effect	SE	LLCI	ULCI
SA PB	Low (2.881)	−0.390	0.044	−0.475	−0.305
	Middle (3.714)	−0.237	0.037	−0.310	−0.163
	High (4.548)	−0.083	0.051	−0.183	0.016
SA FoMO PB	Low (2.881)	−0.233	0.039	−0.313	−0.160
	Middle (3.714)	−0.182	0.024	−0.229	−0.137
	High (4.548)	−0.131	0.028	−0.183	−0.074

SE, Standard Error; SA, SNS Addiction; FoMO, Fear of Missing Out; COM, Competence; PB, Prosocial Behavior.

The moderating role of the need for relatedness on the mediating effect of FoMO was examined in SPSS using the PROCESS macro (Model 15) (Hayes, 2012). The study results indicated that the need for relatedness directly moderated the relationship between SNS addiction and prosocial behavior. There was a notable simple effect when the level of need for relatedness was low (simple effect = −0.393, SE = 0.062, 95% CI [−0.514, −0.272], interval does not contain 0). Conversely, when the level of relatedness was high, the effect remained significant (simple effect = −0.220, SE = 0.044, 95% CI [−0.307, −0.133], interval does not contain 0), confirming Hypothesis H5-3. In addition, the need for relatedness affected the relationship between SNS addiction and prosocial behavior by moderating the influence of FoMO, showing a significant simple effect at low (simple effect = −0.283, SE = 0.045, 95% CI [−0.369, −0.194], interval not containing 0) and high levels of relatedness (simple effect = −0.135, SE = 0.027, 95% CI [−0.185, −0.081], interval does not contain 0), thereby establishing Hypothesis H6-3 (Table 8).

**Table 8:** The moderating effect of relatedness.

Relationships	REL	Effect	SE	LLCI	ULCI
SA PB	Low (2.680)	−0.393	0.062	−0.514	−0.272
	Middle (3.578)	−0.306	0.039	−0.383	−0.230
	High (4.476)	−0.220	0.044	−0.307	−0.133
SA FoMO PB	Low (2.680)	−0.283	0.045	−0.369	−0.194
	Middle (3.578)	−0.209	0.026	−0.259	−0.157
	High (4.476)	−0.135	0.027	−0.185	−0.081

SE, Standard Error; SA, SNS Addiction; FoMO, Fear of Missing Out; REL, Relatedness; PB, Prosocial Behavior.

## 5 General discussion, implications, and limitations and further works

### 5.1 General discussion

The exponential growth of the Internet has allowed it to permeate almost every corner of the world, and for many it affects almost every aspect of daily life. SNS is one of the most widely used Internet applications across all age groups. SNS is used for a variety of reasons such as finding information and inspiration, seeking social interaction, enduring boredom, searching for positive emotions, and escaping negative emotions (Brailovskaia et al., 2020; Shensa et al., 2020). Based on previous studies and Cognitive Resource Theory, Social Comparison Theory, the Image Management Theory and Self-Determination Theory, the present study constructed a moderated mediation model with FoMO as the mediating variable and basic psychological needs satisfaction as the moderating variable under the perspective of consumer psychology to explore the driving of prosocial behaviors in SNS addiction Mechanisms. The questionnaire method was used to confirm the negative predictive effect of SNS addiction on prosocial behavior, the partial mediating role of FoMO between SNS addiction and prosocial behavior, and the basic psychological needs satisfaction significantly moderated the mediating effect of FoMO in the relationship between SNS addiction and prosocial behavior. The findings are useful for helping SNS addicted individuals to reduce negative effects, as well as further enriching research related to prosocial behavior.

First, our findings suggest a negative predictive effect of SNS addiction on prosocial behavior. This is consistent with other studies that have found heavy SNS use to be negatively associated with prosocial behavior (Jenkins et al., 2020) and screen time in childhood to be negatively associated with prosocial behavior (Allen and Vella, 2015). There is currently less research related to the effects of SNS addiction on prosocial behavior. SNS use will be negatively associated with social skills, as frequent use of SNSs may reduce face-to-face contact and deprive opportunities to learn social skills in real life (Kuss and Griffiths, 2017). SNS addicts spend a lot of time on social networks, which directly leads to a reduction in the time they spend on social activities and prosocial behavior in real life. At the same time, excessive attention to information on social networks also distracts their attention, making it difficult for them to focus on real-life problems and needs, which in turn reduces the occurrence of prosocial behavior. Interactive behaviors such as liking, commenting, and following on social networks can provide individuals with immediate emotional satisfaction and a sense of belonging. However, this satisfaction is often virtual and transient, and may replace the real satisfaction and sense of accomplishment that individuals obtain through prosocial behavior in real life. Therefore, SNS addicts may be more inclined to seek emotional satisfaction on social networks and less invested in real-life prosocial behavior.

Second, FoMO has a partial mediating role between SNS addiction and prosocial behavior. FoMO has received widespread attention from researchers in psychology, management, journalism and communication and many other disciplines, and some valuable research results have been achieved. Across cultures, SNS addiction predicts higher levels of depression, anxiety, and stress, as validated in previous studies (Sayeed et al., 2020; Yurdagül et al., 2021; Wang et al., 2021; Wang et al., 2023). Various forms of interpersonal insecurity can inhibit or interfere with prosocial behavior when individuals develop self-doubt, anxiety, social fears, and other psychological problems (Mikulincer and Shaver, 2005; Twenge et al., 2007). With the development of the Internet and the widespread use of social media, the information available to people has become redundant and ever-changing, and individuals may find it difficult to know all the information comprehensively due to the limitations of time and cognitive resources. This diffuse anxiety that may arise when individuals fear that missing out on information will result in their own interests being jeopardized is the FoMO. FoMO individuals spend more attentional resources on acquiring and analyzing information than normal individuals, resulting in a chronic state of resource depletion for FoMO individuals, increasing their moral disengagement (Lanaj et al., 2014), incivility (Welsh and Ordóñez, 2014), and pessimistic expectations of events (Rawn and Vohs, 2011). FoMO negatively impacts subjective well-being, negatively correlates with emotional health and interpersonal health (Stead and Bibby, 2017), reduces an individual’s physiological satisfaction and psychological well-being, and decreases an individual’s engagement in prosocial behavior.

Third, basic psychological needs satisfaction directly moderated the relatedness of SNS addiction to prosocial behavior. High basic psychological needs satisfaction attenuates the effects of SNS addiction on prosocial behavior. Research suggests that individuals who are able to satisfy basic psychological needs satisfaction such as autonomy, competence, and relatedness in a supportive environment will be more inclined to conduct themselves in a pro-social manner (Deci and Ryan, 2008). According to Self-Determination Theory, when humans fulfill the three basic psychological needs of autonomy, competence, and relatedness, they achieve optimal functioning, growth, and well-being, and therefore may experience greater competence to help others autonomously and feel related to others (Ryan et al., 2008). Low basic psychological needs satisfaction enhances the effects of SNS addiction on prosocial behavior. When basic psychological needs satisfaction is not achieved, individuals achieve psychological satisfaction by performing compensatory behaviors and seeking substitutes (Deci and Ryan, 2000). SNS addicts may compensate for their thwarted autonomy, competence, and relatedness needs by becoming more addicted to social networks. According to the attentional resource theory, excessive attention to information on social networks distracts individuals from the outside world, making them have fewer attentional resources for the outside world and decreasing the probability of helpful (prosocial) behaviors.

Fourth, basic psychological needs satisfaction significantly moderated the mediating effect of FoMO in the relationship between SNS addiction and prosocial behavior. High basic psychological needs satisfaction increases prosocial behavior by attenuating the negative effects of FoMO on prosocial behavior. Research suggests that individuals who are highly satisfied with their autonomy, competence, or relatedness needs are better at regulating their emotions, report fewer mental health problems (Moè and Katz, 2021), and have lower levels of perceived FoMO. When the FoMO individual’s needs are met, it increases the individual’s cognitive resources (Stillman et al., 2009), increasing their willingness to engage in prosocial behavior. Low basic psychological needs satisfaction decreases prosocial behavior by enhancing the negative effects of FoMO on prosocial behavior. Research has shown that when individuals are in a situation of basic psychological needs not being satisfied, they will engage in compensatory activities to satisfy basic psychological needs, and maladaptive emotions and behaviors will exacerbate the FoMO situation, increase the depletion of volitional resources, so that the FoMO individual is less likely to give volitional resources to information that does not pertain to him or her, and exacerbate the negative effects of FoMO on prosocial behavior (Xia et al., 2021).

### 5.2 Theoretical and practical implications

Firstly, although previous research has indicated some positive effects of SNS use on adolescent development (Wang et al., 2017; Wang et al., 2019), this study reveals that SNS addiction strongly predicts FoMO and inversely affects prosocial behavior. SNS provide a platform for self-improvement and impression management. According to image management theory, individuals can construct or reconstruct their personal image based on the values associated with their ideal self (Siibak, 2009). SNS users tend to update their status on positive or significant events rather than share worries and negativity. Individuals who have been using SNS for a longer period of time often see extravagant and interesting things displayed by other people, and can believe that life is unfair and that other people are happier with their lives than they are, which then increases a generalized apprehension, suggesting that those SNS-addicted individuals are more likely to suffer from FoMO, which in turn reduces prosocial behavior. Therefore, at the individual level, it is crucial to mitigate FoMO by actively altering cognition or redirecting attention, enhancing self-emotional regulation through nature, pet care, and community or welfare activities, and by seeking support from family members, friends, and teachers to boost positive emotions and self-awareness. At the family level, monitoring and guiding children toward healthy SNS usage and increasing parent–child interaction time are essential. Research demonstrates that a robust parent–child relationship can reduce adolescents’ inclination toward SNS, and parents should be attentive to their psychological needs (Yue et al., 2022), which in turn may decrease the risk of SNS addiction. At the school level, students should be educated about the adverse effects of SNS addiction on health, through health records maintenance, the establishment of psychological counseling services, screening and positive thinking trainings (Brailovskaia and Margraf, 2023), and courses on emotional management and social relationships, to foster a proper life and values perspective, and enhance prosocial behaviors. At the government level, regulatory authorities should educate the public on the dangers of SNS Addiction through diverse channels. Effective prevention measures should include displaying health-related messages or slogans to users who exceed average screen time for their demographic (Jain and Agrawal, 2021).

Secondly, the present study found that both the direct predictive effect of SNS addiction on prosocial behavior and the mediating role of FoMO in the relationship are moderated by basic psychological needs satisfaction. This finding not only deepens the application of basic psychological needs satisfaction in the study of individual mental health and behavior, but also enriches the theoretical development of Self-Determination Theory in the context of social network use. In practical terms, the study offers strategies for managing FoMO and SNS addiction to enhance prosocial behavior. Meaning derived from prosocial behavior yields benefits for social interactions that can alleviate loneliness and promote individual well-being (Maier et al., 2021). Individuals, schools, and society should look for or provide measures to increase individuals’ basic psychological needs satisfaction in order to attenuate the negative effects of SNS addiction on prosocial behavior. At the individual level, it is crucial to develop personal interests and hobbies, maintain an open mindset, and continuously broaden one’s areas of interest; engage more frequently with society, pursue new knowledge and skills, and continually enrich oneself. At the school level, colorful group activities should be organized; emotional education should be emphasized, incorporating Rogers’ principles of ‘student-centered’ learning, ‘respect’, and ‘sincerity’, which are vital for fostering strong interpersonal relationships. At the societal level, high-quality vocational training should be enhanced to improve self-control and societal understanding, allowing individuals to pursue the most suitable career paths; the media should report more on positive prosocial behaviors, conveying uplifting messages to the public by illustrating the emotional experiences of those who help others and by providing positive evaluations of such events. Individuals can satisfactorily meet their basic needs from these three levels in a conducive and free environment.

### 5.3 Limitations and perspectives

Despite some valuable conclusions, this study has several limitations that need addressing in future research. Firstly, the data were collected in a single country; to enhance the generalizability of the findings, the study should be conducted in a variety of cultural contexts. Secondly, this study did not classify the nature of content in SNS addiction, whether it spreads positive or negative messages and its impact on the prosocial behavior of individuals addicted to SNS. Thirdly, future research could investigate if there are variations in the impact of SNS addiction across different dimensions of prosocial behavior such as openness, anonymity, altruism, adherence, emotionality, and urgency. Fourthly, future studies should include additional mediating variables, such as jealousy, social self-awareness, and empathy, to refine the current research model. Fifthly, the study utilized only one moderating variable, basic psychological need satisfaction; subsequent research could explore other relational variables, such as sense of belonging, emotional intelligence, and emotionally regulated self-efficacy, to enhance predictions on the effects of SNS addiction on prosocial behavior. Sixthly, future research could explore whether there are differences in the effects of SNS addiction on online prosocial behavior, and offline prosocial behavior. Seventhly, future research could explore the relatedness between SNS addiction, cyber victimization, basic psychological needs satisfaction, and prosocial behavior.

## 6 Conclusion

The findings of this study suggest that SNS addiction negatively predicts prosocial behavior. FoMO serves as a partial mediator between SNS addiction and prosocial behavior. As individuals increase their usage of SNS, they also heighten their experience of FoMO, which in turn diminishes their prosocial behavior. Basic psychological need satisfaction was found to significantly moderate the mediating effect of FoMO in the relationship between SNS addiction and prosocial behavior. The adverse effects of SNS addiction on prosocial behavior are substantially mitigated when individuals’ basic psychological need satisfaction is high, and conversely, these effects are significantly exacerbated when this satisfaction is low. This study broadens the existing literature on SNS addiction and prosocial behavior while offering management insights applicable to individuals, schools, society, and government.

## Data Availability

The raw data supporting the conclusions of this article will be made available by the authors, without undue reservation.
