# Supplementary material for: Effect of SNS addiction on prosocial behavior: mediation effect of fear of missing out
Source: Front Psychol. 2024 Dec 23;15:1490188. doi: 10.3389/fpsyg.2024.1490188 (PMC11700740; doi:10.3389/fpsyg.2024.1490188)
Supplement: Supplementary file 1 [file Table_1.doc]

Measurement Items

| Variables | Items | Source |
| --- | --- | --- |
| SNS addiction | 1. I have difficulties focusing on my studies or work due to the SNS use.  2. I lose sleep over spending more time on the SNS.  3. This SNS use interferes with doing social activities.  4. My family or friends think that I spend too much time on the SNS.  5. I feel anxious if I cannot access the SNS.  6. I have attempted to spend less time on the SNS but have not succeeded. | Gao, Liu, & Li (2017) |
| Fear of Missing Out | 1. I fear others have more rewarding experiences than me.  2. I fear my friends have more rewarding experiences than me.  3. I get worried when I find out my friends are having fun without me.  4. I get anxious when I don’t know what my friends are up to.  5. It is important that I understand my friend’s ‘‘jokes’’.  6. Sometimes, I wonder if I spend too much time keeping up with what is going on.  7. It bothers me when I miss an opportunity to meet up with friends.  8. When I have a good time, it is important for me to share the details online (e.g., updating status).  9. When I miss out on a planned get-together it bothers me.  10. I keep tabs on what my friends are doing when I go on vacation. | Przybylski et al. (2013) |
| Basic Psychological Need Satisfaction | 1. I feel like I am free to decide for myself how to live my life.  2. I really like the people I interact with.  3. Often, I do not feel very competent.  4. I feel pressured in my life.  5. People I know tell me I am good at what I do.  6. I get along with people I come into contact with.  7. I pretty much keep to myself and don’t have a lot of social contacts.  8. I generally feel free to express my ideas and opinions.  9. I consider the people I regularly interact with to be my friends.  10. I have been able to learn interesting new skills recently.  11. In my daily life, I frequently have to do what I am told.  12. People in my life care about me.  13. Most days I feel a sense of accomplishment from what I do.  14. People I interact with on a daily basis tend to take my feelings into consideration.  15. In my life I do not get much of a chance to show how capable I am.  16. There are not many people that I am close to.  17. I feel like I can pretty much be myself in my daily situations.  18. The people I interact with regularly do not seem to like me much.  19. I often do not feel very capable.  20. There is not much opportunity for me to decide for myself how to do things in my daily life.  21. People are generally pretty friendly towards me. | Johnston & Finney (2010) |
| Prosocial Behavior | 1. I think that one of the best things about helping others is that it makes me look good.  2. I believe that donating goods or money works best when it is tax-deductible.  3. I tend to help others particularly when they are emotionally distressed.  4. I believe I should receive more recognition for the time and energy I spend on charity work.  5. I feel that if I help someone, they should help me in the future. | Carlo & Randall (2002) |
